# Quantifying Waterlogging Tolerance and Recovery Capacity of Jute Genotypes During Early Growth Stage Through Agronomic Trait‐Based Comprehensive Evaluation Value

**DOI:** 10.1155/sci5/8323167

**Published:** 2026-09-08

**Authors:** Rezowana Nizam, Md. Hasanuzzaman, A. M. M. Shamsuzzaman, Mofasser Rahman, Jubayer Al Mahmud, Suraya Parvin, Mohammad Mahbub Islam

**Affiliations:** ^1^ Department of Agricultural Botany, Sher-e-Bangla Agricultural University, Sher-e-Bangla Nagar, Dhaka 1207, Bangladesh, sau.edu.bd; ^2^ Department of Agronomy, Sher-e-Bangla Agricultural University, Sher-e-Bangla Nagar, Dhaka 1207, Bangladesh, sau.edu.bd; ^3^ Department of Agribusiness and Marketing, Sher-e-Bangla Agricultural University, Sher-e-Bangla Nagar, Dhaka 1207, Bangladesh, sau.edu.bd; ^4^ Department of Agroforestry and Environmental Science, Sher-e-Bangla Agricultural University, Sher-e-Bangla Nagar, Dhaka 1207, Bangladesh, sau.edu.bd; ^5^ Bangladesh Agricultural Research Council (BARC), Dhaka 1215, Bangladesh

**Keywords:** biomass, comprehensive approach, *Corchorus olitorius*, genotype, mortality rate, recovery period, seedling, soil waterlogging, trait

## Abstract

Effective screening of genotypes for waterlogging tolerance and recovery capability is critical to mitigate waterlogging stress and support targeted breeding programs. This study was conducted to evaluate 70 tossa jute (*Corchorus olitorius*) genotypes at the seedling stage under 10, 12, and 14 days of waterlogging stress to identify variation in tolerance on the basis of seedling mortality. Fifteen genotypes with contrasting responses were subsequently subjected to detailed agronomic trait analysis under 14 days of continuous waterlogging stress, followed by 14 days of recovery after drainage. Significant genotypic variation was observed in seedling mortality, which increased with increasing stress duration, ranging from 0% to 20% in highly tolerant genotypes to 81%–100% in highly susceptible ones. Waterlogging stress and recovery conditions negatively affected shoot and root length, total length, the root‐to‐shoot ratio, and shoot and root fresh and dry weights. Comprehensive evaluation of waterlogging tolerance (CEVWT) and comprehensive evaluation of recovery tolerance (CEVRT) ranged from 0 to 1.0, reflecting a substantial variation among the genotypes. The waterlogging tolerance coefficient (WTC), CEVWT, waterlogging tolerance score (WTS), recovery tolerance coefficient (RTC), CEVRT, and recovery tolerance score (RTS) suggested that the CVL‐1 and A‐3539 genotypes were identified as highly waterlogging‐tolerant with recovery capability, whereas A‐2521 and O‐9897 were highly sensitive with poor recovery potential. The significant positive correlations of WTS and RTS with different agronomic traits indicated that longer roots and shoots and higher seedling biomass are key contributing traits for waterlogging tolerance and recovery capacity. A significant positive correlation between WTS and RTS demonstrated that genotypes with higher tolerance during waterlogging stress also showed superior recovery potential. Therefore, the findings highlighted that selecting jute genotypes with longer roots and shoots, greater seedling biomass retention under waterlogging stress, and higher recovery after drainage are recommended to develop resilient jute varieties suitable for flood‐prone environments.

## 1. Introduction

Waterlogging, which affects 16% of the cultivated land in the United States, 10% of the agricultural soil of Russia, and the crop growing areas of China, India, Pakistan, and Bangladesh, is one of the most limiting factors that threaten plant growth and development among different abiotic stresses derived from climate change worldwide [1]. The estimated annual financial loss in agriculture exceeds $60 billion due to waterlogging [2]. With an increase in climate change caused by global warming, waterlogging events have become more severe, recurrent, and unpredictable globally [3]. The major limitation of waterlogging is anoxia (absence of O_2_) in soils, causing a decrease in the soil redox potential and severe hypoxia or anoxia within roots [4], severely disrupting the transport of nutrients from roots to shoots, which consequently affects plant growth through mineral nutrient deficiencies or toxicity [5] and reduces yield and quality [6, 7]. Waterlogging stress adversely affects the growth and development of plant seedlings by preventing shoot length, root length, and biomass; limiting nutrient uptake; and decreasing tiller number and yield attributes and yield [8–11]. Waterlogging stress also inhibits photosynthesis by reducing chlorophyll synthesis, transpiration by closing stomata, and respiration of leaves, affecting signal transduction and amino acid metabolism, resulting in leaf senescence and lower accumulation of carbon assimilation, especially when it is induced during the early growth stage of plants [12–15]. Previous studies have revealed that waterlogging stress imposed at the seedling stage significantly decreases the aboveground biomass, plant height, leaf number, leaf area, and SPAD values, leading to decreased yield [16, 17].

Jute, often hailed as the “golden fiber,” is a crucial component of Bangladesh’s agriculture and economy. Jute, which belongs to the Malvaceae family and is scientifically categorized under the genus *Corchorus*, comprises two commercially important species: *Corchorus capsularis* (white jute) and *Corchorus olitorius* (commonly known as tossa jute) [18]. Tossa jute is especially valued for its superior fiber quality, making it a preferred choice for cultivation in many parts of the country. Bangladesh, one of the largest producers of jute, relies heavily on this crop for its rural employment, export earnings, and sustainable agricultural practices [19]. The major jute‐producing regions of Bangladesh include Faridpur, Jashore, Tangail, Munshiganj, and Kushtia. These areas offer favorable agroclimatic conditions such as adequate rainfall, fertile alluvial soils, and a humid environment, which are essential for healthy jute growth and high fiber yield [20]. Jute cultivation also plays a role in preserving the ecological balance because of its biodegradable nature and low environmental footprint compared with those of synthetic fibers [21]. Despite its economic and environmental benefits, jute production is increasingly threatened by the adverse effects of climate change. The anaerobic conditions imposed by waterlogging disrupt normal physiological and metabolic processes in plants, especially during the early seedling stage of jute. The consequences include inhibited root development, chlorosis (yellowing of leaves), stunted growth, and in severe cases, seedling mortality [13]. Prolonged exposure to waterlogged conditions drastically reduces plant vigor and ultimately, the fiber yield of jute [18, 22].

To ensure the sustainability of jute production in the face of such environmental challenges, the development of effective stress management strategies is imperative. One promising avenue is the screening of tossa jute genotypes to evaluate their tolerance or susceptibility to waterlogging conditions [21]. Through such screening programs, genotypes that exhibit superior tolerance can be identified and preserved as valuable genetic resources. These tolerant genotypes can serve as the foundation for future breeding programs aimed at developing high‐yielding, waterlogging‐resistant jute varieties [23]. This approach not only strengthens the resilience of jute cultivation to climate‐induced stresses but also contributes to securing the livelihoods of millions of farmers who depend on this vital crop in India and Bangladesh [24].

The waterlogging tolerance of plants is a complex and comprehensive trait; therefore, developing an effective way to assess the waterlogging tolerance of jute genotypes is vital. The ability of plants to recover from stress is also an important characteristic of plant vitality and survival. Combining the agronomic traits of different jute genotypes under waterlogging stress and recovery after drainage is an effective method for identifying the waterlogging tolerance of different jute genotypes. Previous studies have suggested that selecting the genotypes of crops with the least reduction in agronomic and physiological traits after drainage is constructive for screening many waterlogging genotypes [11, 25, 26].

However, there may be information overlap between different traits, and every single trait may play a diverse role in waterlogging tolerance. Therefore, it is difficult to evaluate the waterlogging tolerance of various genotypes precisely via the use of multiple traits directly. To overcome the lack of single‐trait evaluations of the waterlogging tolerance of genotypes, principal component and membership function analyses followed by comprehensive evaluations of multiple traits of waterlogging genotypes are widely used. Although these multivariate statistical methods have been widely used in the evaluation of plant stress tolerance, including the drought resistance of wheat evaluated by the membership function value (MFV) [27], the salinity tolerance of wheat evaluated and classified by principal component analysis (PCA) and cluster analysis [28], and the waterlogging tolerance of peanuts [11] and wheat identified via the comprehensive evaluation index [10], their application in jute remains limited. More importantly, previous studies on different crops have primarily focused on plant performance under stress conditions, with little attention given to poststress recovery capacity, which is a critical determinant of plant performance stability in environments where stress is transient, such as waterlogging‐prone regions. In jute, the physiological and morphological traits associated with recovery after waterlogging are poorly understood, and no integrative framework currently exists to evaluate both stress tolerance and recovery ability simultaneously. Identifying correlations between waterlogging tolerance, recovery ability, and different agronomic traits is also important for evaluating the response mechanism of different jute genotypes to waterlogging stress and recovery after drainage and exploring reliable comprehensive evaluation methods for identifying jute waterlogging tolerance. The present study addresses this gap by applying MFV and comprehensive evaluation value (CEV) analyses to evaluate jute genotypes under both waterlogging stress and poststress recovery conditions. A key strength of this approach lies in integrating the comprehensive evaluation value for waterlogging tolerance (CEVWT) with that for recovery capacity (CEVRT), providing a more holistic assessment of genotype performance than either measure alone. This combined approach enables the identification of genotypes that not only tolerate waterlogging stress but also recover efficiently once the stress is removed, an attribute that is important for improving crop resilience under fluctuating environmental conditions. In view of this, the main objectives of this study were the simultaneous evaluation of waterlogging tolerance and recovery capacity, the screening of diverse jute genotypes, and the application of integrated multivariate evaluation approaches. Although membership function and comprehensive evaluation approaches have previously been used to assess abiotic stress tolerance in various crops, their integrated application to simultaneously evaluate both waterlogging tolerance and poststress recovery has, to our knowledge, rarely been reported in jute. Accordingly, the present study screened a large number of jute genotypes (*n* = 70) to identify waterlogging‐tolerant genotypes at the early seedling stage, followed by a comprehensive evaluation of 15 selected genotypes under both waterlogging stress and poststress recovery conditions, to support the sustained productivity and quality of jute in Bangladesh under climate change, an outcome that will require combined efforts in genetic improvement, adaptive cultivation practices, and continued research into abiotic stress tolerance.

## 2. Materials and Methods

### 2.1. Plant Growth Conditions

The experiment was conducted at the Department of Agricultural Botany, Sher‐e‐Bangla Agricultural University, Dhaka, Bangladesh, in 2024 to screen tossa jute genotypes to evaluate waterlogging stress tolerance and recovery capacity at the seedling stage. The initial set of 70 genotypes was chosen to represent a broad genetic base of tossa jute, sourced from the Bangladesh Jute Research Institute (BJRI), encompassing diverse breeding lines and released varieties to capture maximum variability in waterlogging response for effective screening. The study comprised three parts: (a) an assessment of the mortality of 70 genotypes under three durations of waterlogging (10, 12, and 14 days); (b) an evaluation of the growth performance of 15 selected genotypes under 14 days of waterlogging stress; and (c) an evaluation of the recovery capability of genotypes after 14 days of drainage. A completely randomized design (CRD) with three replications was used for each experiment. In Experiment (a), 20 seeds per genotype were placed on multilayered moist filter paper in Petri dishes to conduct a germination test under controlled environmental conditions (temperature: 25 ± 2°C; relative humidity: 60%; light intensity: 15,000 lux; photoperiod: 8 h dark and 16 h light). At 7 days after sowing, uniformly emerged seedlings of jute genotypes at the 2‐leaf stage were subjected to experimental treatments consisting of waterlogging and control treatments for 10, 12, and 14 days. Waterlogging treatment was imposed on jute plants by adding regular water containing hydroponic nutrient solutions A and B developed by the Bangladesh Agricultural Research Institute (BARI) (see [29] for details) until the water level filled the Petri dishes to simulate waterlogging conditions. Petri dishes containing jute plants under control (nonwaterlogging) treatments were maintained at optimum field capacity (well‐watered). After 10, 12, and 14 days of the treatment, waterlogged and control plants were evaluated for mortality rate calculated as the percentage of dead seedlings among the germinated population. In Experiment (b), 15 genotypes were selected from Experiment (a) based on multiple criteria, including contrasting mortality under prolonged stress (14 days), consistency of response across stress durations (10–14 days), phenotypic diversity, and agronomic relevance incorporating both widely cultivated varieties and advanced breeding lines. Seeds were grown in small plastic pots (10 × 4 × 4 cm^3^) filled with the pot mixture containing 75% field soil, 10% coarse sand, 10% vermicompost, and 5% cocopeat. Fifteen seeds of each genotype were sterilized with 5% sodium hypochlorite and thoroughly rinsed with tap water, and then sown in the pots. After germination, five seedlings of similar size were kept in each pot at the two‐leaf stage. Waterlogging treatment was imposed at the four‐leaf stage, and the water level was maintained 3 cm above the soil surface during the waterlogging process for 14 days. The control group of each jute genotype was grown under normal irrigated conditions, maintaining the soil moisture at 65%–70% of the water‐holding capacity. The water in the pots was drained after waterlogging stress for 14 days to evaluate the recovery capacity of the same 15 jute genotypes in the Experiment (c).

### 2.2. Measurement of Seedling Mortality Percentage

The mortality rate is typically calculated as the number of dead seedlings divided by the total population or sample size, expressed as a percentage.

### 2.3. Measurement of Agronomic Traits

After 14 days of waterlogging stress and recovery after drainage, five seedlings from each plastic pot were selected to determine the shoot, root, and total length of individual seedlings via a measuring scale, and the average value in centimeters was recorded. The root‐to‐shoot ratio was determined by the ratio of root length to shoot length. To determine the fresh and dry biomass of shoots and roots, five seedlings from each pot were uprooted without disturbing the root system and gently washed with tap water until all the adhering particles of the pot mixture were washed away from the root system. Excess water was wiped out by placing it between the folds of blotting paper to achieve accurate estimations, and then, the seedlings were cut at the collar region to divide them into roots and shoots. The fresh root weight and fresh shoot weight were subsequently recorded via an electric balance. The harvested samples were then oven‐dried at 70 ± 2°C for 72 h until a constant weight was achieved. After drying, the dry weights of the shoots and roots were recorded via an electric balance.

### 2.4. Calculation of the CEVWT and CEVRT

The waterlogging tolerance coefficient (WTC) and recovery tolerance coefficient (RTC) of all agronomic traits were calculated as the ratio of waterlogging stress to the control value and recovery treatment to the control value for each trait, respectively.

The MFV of each genotype was determined via the following equation (Equation (1)):

(1) uXg=Xg−XminXmax−Xmin g=123, , ,……n.

where *u*(*X*_*g*_) represents the MFV of the *g*th comprehensive indicator, *X*_*g*_ represents the *g*th comprehensive indicator value, *X*_max_ represents the maximum value of the *g*th comprehensive indicator, and *X*_min_ represents the minimum value of the *g*th comprehensive indicator.

The index weight of each comprehensive indicator was calculated via the following equation (Equation (2)):

(2) Wg=Pg ∑g=1nPg , g=123, , ,…,n,

where *W*_*g*_ is the index weight of the *g*th comprehensive indicator among all comprehensive indicators and *P*_*g*_ is the contribution rate of the *g*th comprehensive indicator.

The CEV of each genotype was determined via the following equation (Equation (3)):

(3) CEV=∑gnuXg×Wgg=123, , ,…,n,

where CEV indicates the waterlogging and/or recovery tolerance of jute genotypes. The higher the CEV value is, the greater the tolerance, and the lower the CEV value is, the lower the tolerance to waterlogging stress and/or recovery of jute genotypes.

PCA of the relative changes (% control) in all of the agronomic traits of the different jute genotypes during the waterlogging and recovery stages was performed to determine the comprehensive components.

### 2.5. Statistical Analysis

SPSS 22.0 was used for statistical analysis of variance (ANOVA), PCA, and cluster analysis; Microsoft Excel 2010 was used for Pearson’s correlation. All data are means (±SE) of three replications. Prior to analysis, the assumptions of normality and homogeneity of variance were evaluated using the Shapiro–Wilk test and Levene’s test, respectively, based on the residuals of the ANOVA model. The residuals satisfied the assumptions of normality and homogeneity of variance for all measured traits (*p* > 0.05). Multiple comparison range tests were performed via the DMRT test. The degree of correlation among different variables was determined via Pearson’s correlation coefficient (*r*) analysis and correlation coefficient matrix analysis. *p* < 0.05, *p* < 0.01, and *p* < 0.001 were considered statistically significant.

## 3. Results

### 3.1. Seedling Mortality on Different Days of Waterlogging Stress

**FIGURE 1:**
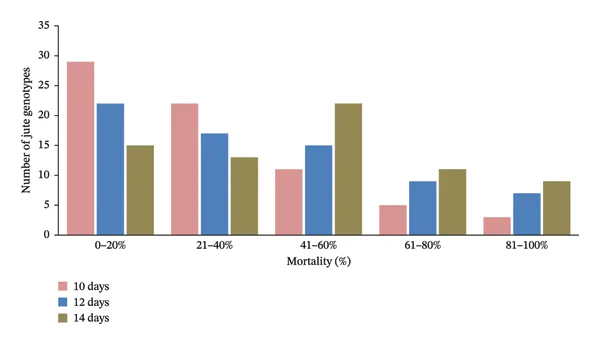
Jute genotypes with varying mortality rates at 10, 12, and 14 days of durations of waterlogging stress at seedling stage.

Waterlogging restricts oxygen availability to roots, inducing hypoxia and increasing seedling mortality. To assess tolerance among 70 jute genotypes, seedling mortality was recorded after 10, 12, and 14 days of waterlogging, and genotypes were grouped into five mortality categories (0%–20%, 21%–40%, 41%–60%, 61%–80%, and 81%–100%; Figure 1, Supporting Table 1). This classification helps to reflect survival ability under waterlogging stress and simplifies data interpretation, enabling the identification of genotypes with varying waterlogging tolerance levels for the next experiment.

Mortality increased with increasing stress duration. The number of waterlogging tolerant jute genotypes in the lowest mortality category (0%–20%) declined from 29 at 10 days to 22 at 12 days and to only 15 at 14 days, while the number of highly sensitive genotypes (81%–100% mortality) rose correspondingly over the same period. Based on their consistently low mortality (0%–20%) after 14 days of waterlogging, 15 genotypes (A‐1119, TP‐8, A‐1362, A‐3539, A‐1216, CVL‐1, A‐2521, TP‐3, A‐1770, A‐2446, O‐9897, A‐3343, JRO‐524, A‐1221, and A‐1436) were selected for detailed agronomic evaluation. These studies collectively demonstrate that waterlogging tolerance differs widely among genotypes, with direct consequences for plant survival and resilience under prolonged waterlogged conditions.

### 3.2. Performance of Genotypes on the Basis of Agronomic Traits

**FIGURE 2:**
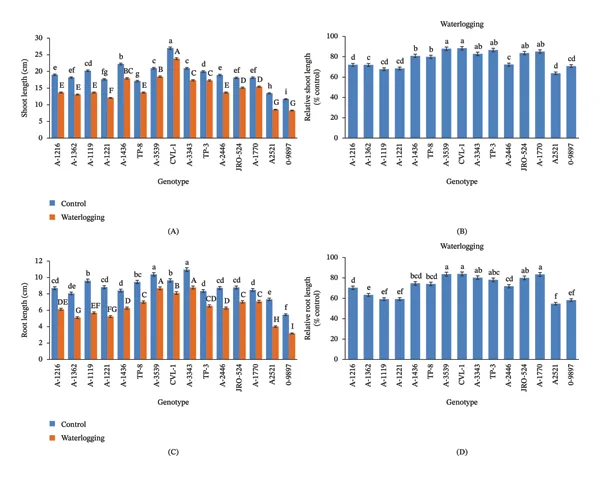
Effect of control and 14 days of waterlogging stress on (A) shoot length, (B) relative shoot length (% control), (C) root length, and (D) relative root length (% control) of 15 jute varieties. Data represent the mean ± SE calculated by three replications. Small letters on bars represent statistically significant differences (*p* < 0.01) within the varieties under control and waterlogging treatment as determined by the DMRT.

**FIGURE 3:**
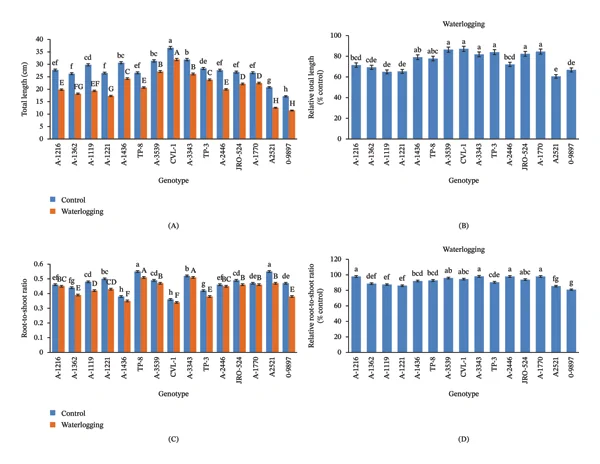
Effect of control and 14 days of waterlogging stress on (A) total length, (B) relative total length (% control), (C) root‐to‐shoot ratio, and (D) relative root‐to‐shoot ratio (% control) of 15 jute varieties. Data represent the mean ± SE calculated by three replications. Small letters on bars represent statistically significant differences (*p* < 0.01) within the varieties under control and waterlogging treatment as determined by the DMRT.

**FIGURE 4:**
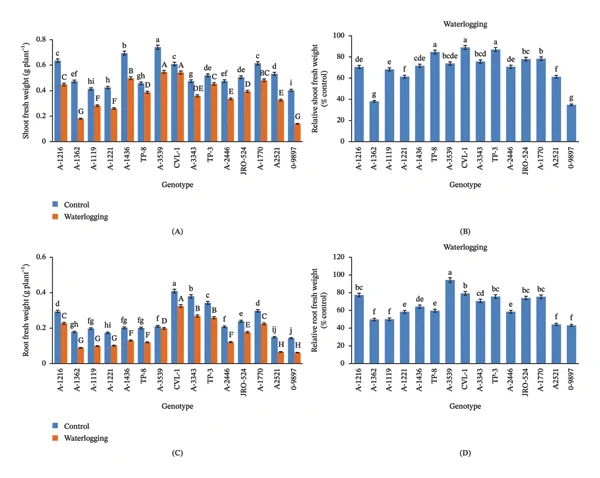
Effect of control and 14 days of waterlogging stress on (A) shoot fresh weight, (B) relative shoot fresh weight (% control), (C) root fresh weight, and (D) relative root fresh weight (% control) of 15 jute varieties. Data represent the mean ± SE calculated by three replications. Small letters on bars represent statistically significant differences (*p* < 0.01) within the varieties under control and waterlogging treatment as determined by the DMRT.

**FIGURE 5:**
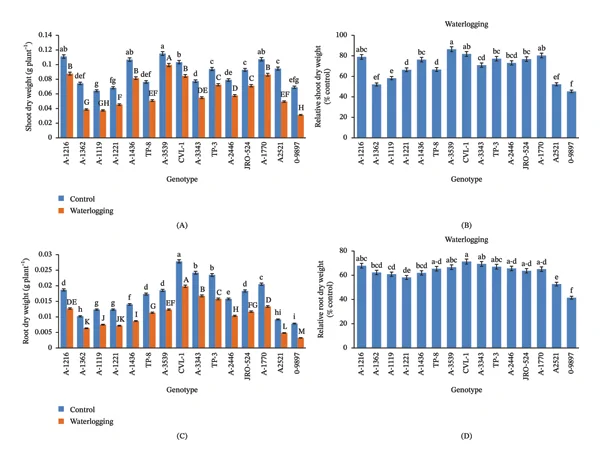
Effect of control and 14 days of waterlogging stress on (A) shoot dry weight, (B) relative shoot dry weight (% control), (C) root dry weight, and (D) relative root dry weight (% control) of 15 jute varieties. Data represent the mean ± SE calculated by three replications. Small letters on bars represent statistically significant differences (*p* < 0.01) within the varieties under control and waterlogging treatment as determined by the DMRT.

Waterlogging significantly reduced all growth‐related traits, including shoot length, root length, total seedling length, root‐to‐shoot ratio, shoot fresh weight, root fresh weight, shoot dry weight, and root dry weight across the 15 selected jute genotypes under 14 days of waterlogging stress (Figures 2–5), although the extent of reduction varied by growth and biomass traits and genotype. Root, shoot, and total seedling length were comparatively less affected, retaining approximately 59.3%–88.3% of control values under 14 days of waterlogging stress conditions. Fresh and dry biomass traits were more sensitive and reduced by 2–3‐fold, retaining relative values of 34.8%–94.4% of control values depending on genotypes under waterlogging conditions. The root‐to‐shoot ratio was the least affected trait (80.9%–98.1% of control), indicating that root and shoot growth were suppressed proportionally rather than through a shift in biomass allocation. Across nearly all growth and biomass traits, CVL‐1 and A‐3539 consistently retained the highest relative values (expressed as % control) of growth and biomass under waterlogging, whereas O‐9897, A‐1221, and A‐2521 showed the highest reductions, identifying these genotypes as tolerant and sensitive to waterlogging stress, respectively (Figures 2–5).

**FIGURE 6:**
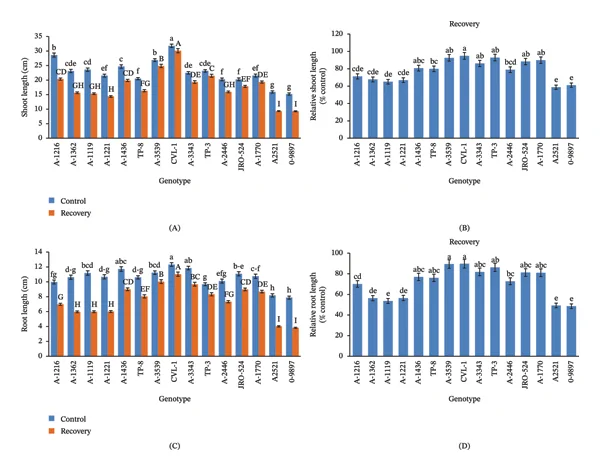
Effect of control and 14 days of recovery after drainage on (A) shoot length, (B) relative shoot length (% control), (C) root length, and (D) relative root length (% control) of 15 jute varieties. Data represent the mean ± SE calculated by three replications. Small letters on bars represent statistically significant differences (*p* < 0.01) within the varieties under control and waterlogging treatment as determined by the DMRT.

**FIGURE 7:**
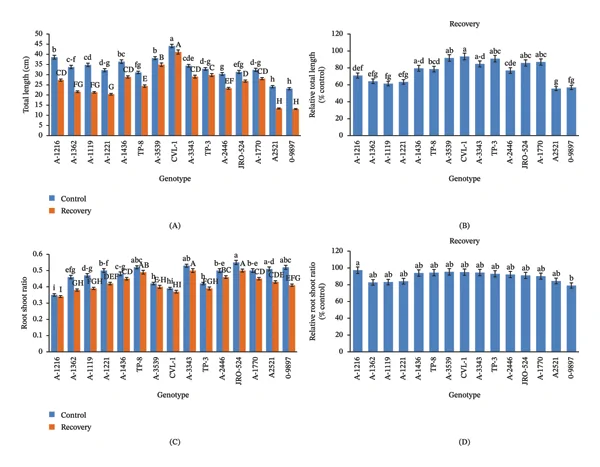
Effect of control and 14 days of recovery after drainage on (A) total length, (B) relative total length (% control), (C) root‐to‐shoot ratio, and (D) relative root‐to‐shoot ratio (% control) of 15 jute varieties. Data represent the mean ± SE calculated by three replications. Small letters on bars represent statistically significant differences (*p* < 0.01) within the varieties under control and waterlogging treatment as determined by the DMRT.

**FIGURE 8:**
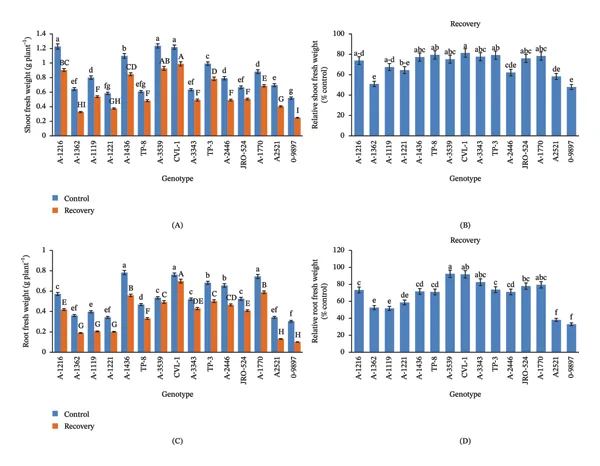
Effect of control and 14 days of recovery after drainage on (A) shoot fresh weight, (B) relative shoot fresh weight (% control), (C) root fresh weight and (D) relative root fresh weight (% control) of 15 jute varieties. Data represent the mean ± SE calculated by three replications. Small letters on bars represent statistically significant differences (*p* < 0.01) within the varieties under control and waterlogging treatment as determined by the DMRT.

**FIGURE 9:**
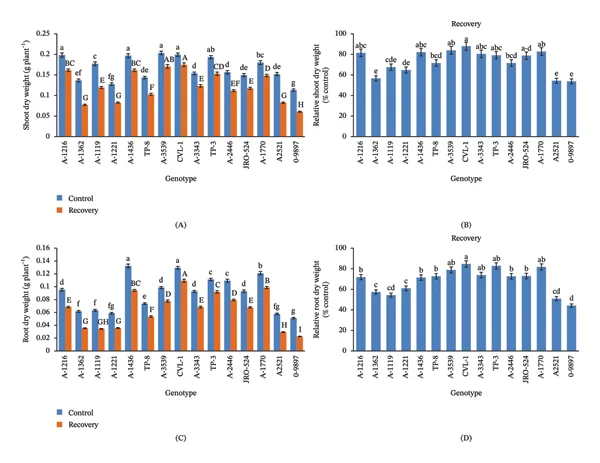
Effect of control and 14 days of recovery after drainage on (A) shoot dry weight, (B) relative shoot dry weight (% control), (C) root dry weight, and (D) relative root dry weight (% control) of 15 jute varieties. Data represent the mean ± SE calculated by three replications. Small letters on bars represent statistically significant differences (*p* < 0.01) within the varieties under control and waterlogging treatment as determined by the DMRT.

Following 14 days of drainage after waterlogging stress, all genotypes showed partial recovery of growth and biomass, but recovery capacity again differed substantially (Figures 6–9). The root, shoot, and total seedling length traits recovered to about 48.6%–94.9% of control values and biomass traits to about 33.0%–92.5%, while the root‐to‐shoot ratio remained comparatively stable (79%–97.3%), reinforcing its limited value for distinguishing recovery capacity. CVL‐1 and A‐3539 again showed the strongest recovery of growth and biomass, whereas O‐9897, A‐2521, and A‐1221 recovered most poorly, reflecting their performance during the waterlogging stress phase. These results indicate that genotypes with greater waterlogging tolerance also possess stronger poststress recovery capacity.

### 3.3. Performance of Genotypes on the Basis of the WTC and RTC

Substantial genotypic variation was observed in both WTC and RTC (Table 1). After 14 days of waterlogging, genotype A‐3539 and CVL‐1 had the highest mean WTC (0.84), followed by TP‐3 and A‐1770 (0.81), indicating superior tolerance to waterlogging stress. In contrast, genotype O‐9897 (0.55) and A‐2521 (0.59) had the lowest mean WTC, reflecting higher sensitivity to waterlogging stress. After 14 days of drainage, genotype CVL‐1 (0.90) and A‐3539 (0.87) had the highest mean RTC, followed by TP‐3 (0.85) and A‐1770 (0.84), while genotype O‐9897 (0.53) and A‐2521 (0.56) again ranked the lowest RTC. Higher WTC and RTC values were consistently associated with greater tolerance and recovery ability, and lower values with greater susceptibility.

**TABLE 1:** Waterlogging tolerance coefficient (WTC) and waterlogging recovery coefficient (WRC) of agronomic traits of jute genotypes after 14 days of waterlogging and drainage recovery.

Genotype	A‐1216	A‐1362	A‐1119	A‐1221	A‐1436	TP‐8	A‐3539	CVL‐1	A‐3343	TP‐3	A‐2446	JRO‐524	A‐1770	A‐2521	O‐9897
SL (W)	0.72	0.72	0.68	0.68	0.81	0.80	0.88	0.88	0.83	0.87	0.72	0.84	0.85	0.64	0.71
RL (W)	0.70	0.63	0.59	0.59	0.75	0.74	0.84	0.84	0.80	0.78	0.72	0.80	0.83	0.55	0.58
TL (W)	0.71	0.69	0.65	0.65	0.79	0.78	0.86	0.87	0.82	0.84	0.72	0.82	0.85	0.61	0.67
RSR (W)	0.98	0.89	0.88	0.86	0.92	0.93	0.96	0.94	0.98	0.90	0.98	0.94	0.98	0.85	0.81
SFW (W)	0.71	0.38	0.68	0.61	0.72	0.85	0.74	0.89	0.76	0.87	0.71	0.78	0.78	0.61	0.35
RFW (W)	0.77	0.50	0.50	0.58	0.64	0.60	0.94	0.79	0.71	0.76	0.59	0.74	0.75	0.44	0.43
SDW (W)	0.79	0.52	0.58	0.66	0.76	0.67	0.86	0.82	0.71	0.77	0.73	0.77	0.80	0.52	0.45
RDW (W)	0.68	0.62	0.61	0.58	0.62	0.65	0.67	0.71	0.69	0.67	0.66	0.64	0.65	0.53	0.41
Average WTC	0.76	0.62	0.65	0.65	0.75	0.75	0.84	0.84	0.79	0.81	0.73	0.79	0.81	0.59	0.55
SL (R)	0.71	0.68	0.65	0.67	0.81	0.80	0.92	0.95	0.86	0.93	0.79	0.88	0.90	0.59	0.61
RL (R)	0.70	0.56	0.53	0.56	0.77	0.76	0.89	0.90	0.82	0.86	0.73	0.81	0.81	0.49	0.48
TL (R)	0.71	0.64	0.61	0.63	0.79	0.78	0.92	0.93	0.85	0.91	0.77	0.86	0.87	0.55	0.57
RSR (R)	0.97	0.83	0.83	0.84	0.94	0.94	0.95	0.95	0.94	0.93	0.92	0.91	0.90	0.84	0.79
SFW (R)	0.74	0.51	0.67	0.64	0.77	0.79	0.75	0.81	0.77	0.79	0.62	0.76	0.78	0.58	0.48
RFW (R)	0.73	0.52	0.52	0.59	0.71	0.71	0.92	0.92	0.82	0.74	0.71	0.78	0.79	0.38	0.33
SDW (R)	0.81	0.56	0.67	0.65	0.82	0.71	0.84	0.88	0.80	0.79	0.71	0.79	0.83	0.54	0.54
RDW (R)	0.72	0.57	0.54	0.61	0.71	0.73	0.79	0.85	0.74	0.83	0.73	0.73	0.82	0.51	0.44
Average WRC	0.76	0.61	0.63	0.65	0.79	0.78	0.87	0.90	0.83	0.85	0.75	0.81	0.84	0.56	0.53

Abbreviations: RDW, root dry weight; RFW, root fresh weight; RL, root length; R, recovery for 14 days after drainage; RSR, root‐to‐shoot ratio; SDW, shoot dry weight; SFW, shoot fresh weight; SL, shoot length; TL, total length; W, waterlogging for 14 days.

### 3.4. Relationships Among Different Agronomic Traits Related to Waterlogging and Recovery After Drainage

Relative (expressed as % of control) values of shoot length, root length, total length, shoot fresh weight, root fresh weight, shoot dry weight and root dry weight were significantly positively correlated with genotype performance under both waterlogging and recovery (Supporting Figures 1–4; Table 2). Relative (expressed as % of control) values of shoot length, root length, total length, shoot fresh weight, root fresh weight, shoot dry weight and root dry weight were significantly positively correlated with genotype performance under both 14 days of waterlogging and recovery conditions (Supporting Figures 1–4; Table 2). The correlation matrix (Table 3) showed strong positive correlation between shoot length and total length (*r* = 0.99 and 0.99, respectively), root length and total length (*r* = 0.91 and 0.95, respectively), shoot fresh and dry weight (*r* = 0.94 and 0.98, respectively), and root fresh and dry weight (*r* = 0.97 and 0.99, respectively) under both 14 days of waterlogging and recovery conditions. In contrast, shoot length was negatively correlated with the root‐to‐shoot ratio (*r* = −0.26 and −0.20) under both waterlogging and recovery. Because of strong positive and negative correlations among the different traits related to waterlogging and recovery, no single indicator could reliably capture overall tolerance or recovery capacity, justifying the use of PCA for a comprehensive evaluation.

**TABLE 2:** Pearson’s correlation analysis between agronomic traits and relative change (% control) measured under control and 14 days of waterlogging and recovery after drainage.

Parameter	SL	RL	TL	RSR	SFW	RFW	SDW	RDW
Pearson’s correlation coefficient (r)								
Control	0.62^∗^	0.58^∗^	0.65^∗∗^	0.12	0.39	0.65^∗∗^	0.73^∗∗^	0.82^∗∗∗^
Waterlogging	0.82^∗∗∗^	0.89^∗∗∗^	0.87^∗∗∗^	0.37	0.83^∗∗∗^	0.82^∗∗∗^	0.91^∗∗∗^	0.85^∗∗∗^
Control	0.53^∗^	0.63^∗^	0.60^∗^	0.41	0.55^∗^	0.74^∗∗^	0.79^∗∗^	0.86^∗∗∗^
Recovery	0.84^∗∗∗^	0.95^∗∗∗^	0.90^∗∗∗^	0.12	0.73^∗∗^	0.89^∗∗∗^	0.93^∗∗∗^	0.92^∗∗∗^

Abbreviations: RDW, root dry weight; RFW; root fresh weight; RL, root length; RSR, root‐to‐shoot ratio; SDW, shoot dry weight; SFW, shoot fresh weight; SL, shoot length; TL, total length.

^∗^A significant difference (*p* < 0.05).

^∗∗^A significant difference (*p* < 0.01).

^∗∗∗^A significant difference (*p* < 0.001).

**TABLE 3:** Correlation coefficient matrix among all agronomic traits of jute after 14 days of waterlogging and recovery after drainage.

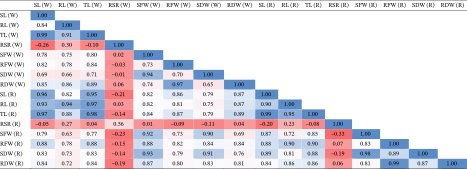

*Note:* Blue for strong positive correlation, red for strong negative correlation, and white for zero correlation.

Abbreviations: R, recovery for 14 days after drainage; RDW, root dry weight; RFW; root fresh weight; RL, root length; RSR, root‐to‐shoot ratio; SDW, shoot dry weight; SFW, shoot fresh weight; SL, shoot length; TL, total length; W, waterlogging for 14 days.

### 3.5. Identification of Waterlogging and Recovery Tolerance Among Jute Genotypes via PCA, Membership Function Analysis, and Comprehensive Evaluation Value

PCA was conducted on the basis of the relative change (% of control) of different agronomic traits of 15 jute genotypes after 14 days of waterlogging and subsequent recovery of drainage conditions, explaining 88.1% and 91.0% of total variance, respectively (Supporting Tables 2–3; Tables 4 and 5). The PCA analysis was consistent with the strong trait inter‐correlation described above in the correlation heatmap (Table 3) which indicating that a dominant axis of variation among the traits and could be used for further analysis. The strong positive correlations among most of the growth and biomass‐related traits those are biologically interdependent, suggesting a high degree of redundancy. The weight of the comprehensive index was 1.0.

**TABLE 4:** Value of each score of the comprehensive indicators (CIx), membership function value (MFV), comprehensive evaluation value of waterlogging tolerance (CEVWT), and waterlogging tolerance score (WTS) of different jute varieties and their calculation under 14 days of waterlogging stress.

Varieties	Score of the comprehensive indicators	MFV	CEVWT	WTS
	Cl1	*u* (X1)		
A‐1216	0.2996	0.7051	0.7051	7
A‐1362	−1.0092	0.2805	0.2805	13
A‐1119	−0.9459	0.3010	0.3010	12
A‐1221	−0.8869	0.3202	0.3202	11
A‐1436	0.2394	0.6856	0.6856	8
TP‐8	0.2173	0.6784	0.6784	9
A‐3539	1.2086	1.0000	1.0000	1
CVL‐1	1.2025	0.9980	0.9980	2
A‐3343	0.7042	0.8364	0.8364	5
TP‐3	0.7605	0.8546	0.8546	4
A‐2446	0.0440	0.6222	0.6222	10
JRO‐524	0.6403	0.8156	0.8156	6
A‐1770	0.9290	0.9093	0.9093	3
A‐2521	−1.5295	0.1117	0.1117	14
O‐9897	−1.8737	0.0000	0.0000	15

*Note:* “CI” values of each genotype were determined by principal component analysis (PCA) and the scores of the comprehensive indicators.

**TABLE 5:** Value of each score of the comprehensive indicators (CIx), membership function value (MFV), comprehensive evaluation value of the recovery tolerance (CEVRT), and recovery tolerance score (RTS) of different jute varieties and their calculation under 14 days recovery after drainage.

Varieties	Component score	MFV	CEVRT	RTS
	Cl1	*u* (X1)		
A‐1216	0.2336	0.6630	0.6630	9
A‐1362	−1.1777	0.2103	0.2103	13
A‐1119	−0.9734	0.2759	0.2758	12
A‐1221	−0.8268	0.3229	0.3229	11
A‐1436	0.4415	0.7296	0.7296	7
TP‐8	0.3369	0.6961	0.6961	8
A‐3539	1.0728	0.9321	0.9321	2
CVL‐1	1.2845	1.0000	1.0000	1
A‐3343	0.7024	0.8133	0.8133	5
TP‐3	0.8797	0.8702	0.8702	3
A‐2446	0.0316	0.5982	0.5982	10
JRO‐524	0.5754	0.7726	0.7726	6
A‐1770	0.7619	0.8324	0.8324	4
A‐2521	−1.5090	0.1040	0.1040	14
O‐9897	−1.8334	0.0000	0.0000	15

Membership function analysis was used to derive a CEVWT and CEVRT for each genotype (Equations (1)–(3)). Genotype O‐9897 had the lowest CEVWT and CEVRT, while genotype A‐3539 had the highest CEVWT and CVL‐1 the highest CEVRT, confirming these genotypes as sensitive and tolerant to waterlogging stress to waterlogging stress, respectively. Genotypes were subsequently ranked 1 (most tolerant/best recovery) to 15 (most sensitive/poorest recovery) to generate a waterlogging tolerance score (WTS) and a recovery tolerance score (RTS).

### 3.6. Correlations of the Relative Percentages of Agronomic Traits With WTS and RTS

**FIGURE 10:**
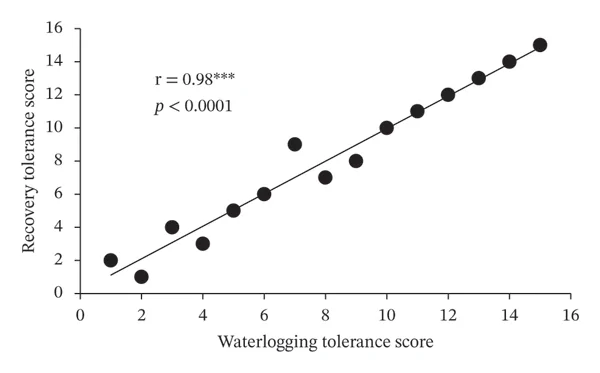
Correlation (Pearson’s r value) between the waterlogging tolerance score and recovery tolerance score under 14 days of waterlogging stress and recovery after drainage on jute varieties. Significant differences for a two‐tailed test are indicated: ∗∗∗, *p* < 0.001.

The relative (% of control) values of shoot length, root length, total seedling length, root‐to‐shoot ratio, shoot fresh weight, root fresh weight, shoot dry weight, and root dry weights were all significantly positively correlated with WTS (*r* = 0.90, 0.94, 0.93, 0.75, 0.77, 0.96, 0.92, and 0.77, respectively) and with RTS (*r* = 0.97, 0.97, 0.97, 0.80, 0.93, 0.92, 0.92, and 0.94, respectively) (Supporting Figs. 5–8; Table 6). A strong positive correlation was also found between WTS and RTS (*r* = 0.98; Figure 10), indicating that waterlogging tolerance during stress strongly predicted subsequent recovery capacity. Lower WTS and RTS values indicate the greater tolerance and recovery capacity, and higher values indicate the greater sensitivity and the weaker recovery.

**TABLE 6:** Pearson’s correlation (*r*) analysis of relative change (% control) of agronomic traits with waterlogging tolerance score (WTS) and recovery tolerance score (RTS) after 14 days of waterlogging and recovery after drainage.

Parameter	SL (%control)	RL (% control)	TL (% control)	RSR (% control)	SFW (% control)	RFW (% control)	SDW (% control)	RDW (% control)
Pearson’s correlation coefficient (r)								
WTS	0.90^∗∗∗^	0.94^∗∗∗^	0.93^∗∗∗^	0.75^∗∗^	0.77^∗∗^	0.96^∗∗∗^	0.92^∗∗∗^	0.77^∗∗^
RTS	0.97^∗∗∗^	0.97^∗∗∗^	0.97^∗∗∗^	0.78^∗∗^	0.80^∗∗^	0.93^∗∗∗^	0.92^∗∗∗^	0.94^∗∗∗^

Abbreviations: RDW, root dry weight; RFW; root fresh weight; RL, root length; RSR, root‐to‐shoot ratio; SDW, shoot dry weight; SFW, shoot fresh weight; SL, shoot length; TL, total length.

^∗∗^A significant difference (*p* < 0.01).

^∗∗∗^A significant difference (*p* < 0.001).

### 3.7. Cluster Analysis of Different Jute Genotypes Based on the CEVWT and CEVRT

**FIGURE 11:**
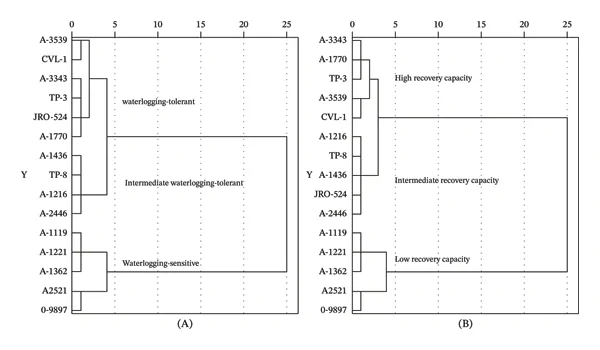
Hierarchical cluster analysis based on the (A) CEVWT value of 14 days of waterlogging stress to evaluate the waterlogging tolerance for 15 jute genotypes and (B) CEVRT value for 14 days of recover after drainage to evaluate the waterlogging recovery capability of 15 jute genotypes.

Hierarchical cluster analysis based on the CEVWT values grouped the 15 genotypes into three distinct classifications of waterlogging tolerance (Figure 11(A)): waterlogging‐tolerant (A‐3539, CVL‐1, A‐1770, TP‐3, A‐3343, and JRO‐524), intermediate waterlogging‐tolerant (A‐1216, A‐1436, TP‐8, and A‐2446) and waterlogging‐sensitive (A‐1221, A‐1119, A‐1362, A‐2521, and O‐9897). As shown in Figure 11B, the 15 genotypes of jute were grouped into three classifications of waterlogging recovery capacity based on CEVRT, such as genotypes with high recovery capacity (A‐3539, CVL‐1, TP‐3, A‐1770, and A‐3343), intermediate recovery capacity (JRO‐524, A‐1436, TP‐8, A‐1216, and A‐2446) and low recovery capacity (A‐2521, O‐9897, A‐1221, A‐1119, and A‐1362).

### 3.8. Categories of Different Jute Genotypes on the Basis of WTS and RTS

Based on WTS and RTS rankings (Table 7), A‐3539 and CVL‐1 ranked highest for both waterlogging tolerance and recovery capacity, while O‐9897 and A‐2521 ranked lowest for both. The 15 genotypes were classified into six waterlogging tolerance categories: highly tolerant (CVL‐1 and A‐3539), tolerant (A‐1770 and TP‐3), moderately tolerant (A‐3343, JRO‐524, and A‐1216), moderately sensitive (A‐1436, TP‐8, and A‐2446), sensitive (A‐1221, A‐1119, and A‐1362), and highly sensitive (A‐2521 and O‐9897). The 15 jute genotypes were further classified into six categories: higher recovery capacity (CVL‐1 and A‐3539), high recovery capacity (A‐1770 and TP‐3), moderate recovery capacity (A‐3343, JRO‐524, and A‐1436), moderately low recovery capacity (TP‐8, A‐1216, and A‐2446), low recovery capacity (A‐1221, A‐1119, and A‐1362) and lower recovery capacity (A‐2521 and O‐9897).

**TABLE 7:** Categories of jute genotypes based on waterlogging tolerance score (WTS) and recovery tolerance score (RTS) under 14 days of waterlogging stress and recovery after drainage to evaluate the waterlogging tolerance and recovery capacity of 15 jute genotypes.

Categories	WTS range to evaluate waterlogging tolerance	Jute genotypes	Categories	RTS range to evaluate recovery capacity	Jute genotypes
Highly tolerant	1–2	CVL‐1, A‐3539	Higher recovery capacity	1–2	A‐3539, CVL‐1
Tolerant	3–4	A‐1770, TP‐3	High recovery capacity	3–4	TP‐3, A‐1770
Moderately tolerant	5–7	A‐3343, JRO‐524, A‐1216	Moderate recovery capacity	5–7	A‐3343, JRO‐524, A‐1436
Moderately sensitive	8–10	A‐1436, TP‐8, A‐2446	Moderately low recovery capacity	8–10	TP‐8, A‐1216, A‐2446
Sensitive	11–13	A‐1221, A‐1119, A‐1362	Low recovery capacity	11–13	A‐1221, A‐1119, A‐1362
Highly sensitive	14–15	A‐2521, O‐9897	Lower recovery capacity	14–15	A‐2521, O‐9897

## 4. Discussions

All jute genotypes exhibited a marked increase in seedling mortality and a decrease in various agronomic traits under waterlogging stress. These results are consistent with previous findings on different plant species [30–33]. When plants are exposed to waterlogging stress, they experience anoxia (absence of O_2_) or hypoxia (reduced O_2_ availability) in the rhizosphere, severely restricting oxygen diffusion to roots and impairing aerobic respiration [34]. Consequently, ATP production declines, limiting energy‐intensive processes essential for growth and survival. The extent of damage is strongly genotype‐dependent and reflects the ability to activate adaptive mechanisms such as internal oxygen transport, aerenchyma development in roots, and anaerobic respiration [30].

Waterlogging imposed at the seedling stage exhibited significant variations in genotype tolerance that have been reported across a wide range of plant species. For example, exposure of 154 maize lines to six days of waterlogging in both pot and field conditions revealed marked differences in waterlogging tolerance and susceptibility in terms of root and shoot traits [35]. Similarly, 12 soybean genotypes exhibited substantial variation in plant growth, biomass, yield contributing traits, and yield under 12 days of waterlogging stress [31]. In barley, 33 genotypes of diverse origins showed contrasting levels of tolerance, enabling the identification of both tolerant and susceptible genotypes [36]. Comparable genotypic variability has also been documented in wheat [37], maize [38], mungbean [39], rapeseed [40], cotton [41], cowpea [42], and other plant species [43], highlighting the widespread genetic diversity in plant responses to waterlogging stress.

Under continuous waterlogging stress, the dissolved oxygen in the water is depleted, resulting in anoxic soil and severe hypoxia or anoxia within roots, and the root respiration of the plant gradually becomes impaired, which may be associated with lower ATP and excess lactic acid production, followed by accumulation of reactive oxygen species (ROS), which could contribute to programmed cell death in the roots, blocked nutrient uptake and transport, and weakened photosynthetic capacity, ultimately affecting plant growth [44–46]. It has been reported that root water uptake is reduced as a result of lower stomatal conductance due to stomatal closure, which disrupted photosynthetic processes and decreased CO_2_ assimilation under waterlogging stress, collectively contributing to decreased shoot and root growth [47]. Waterlogging stress may be associated with severe reduction in the soil redox potential, and increasing the availability of micronutrients by several folds thus leading to their accumulation in shoots at toxic levels [5]. When these micronutrients enter the plant shoot through transpiration flow, this could generate ROS in plant tissue and damage the photosynthetic apparatus, with chlorophyll degradation thus enhancing leaf senescence and restricting plant growth and development [48].

Under waterlogging conditions, tolerant jute genotypes may adopt a quiescence‐type adaptive strategy, characterized by suppression of growth and metabolic activity to conserve energy during stress. This is evident from the reduced shoot and root growth observed across genotypes, which may represent an adaptive response rather than merely stress‐induced damage. By limiting growth, tolerant genotypes may preserve carbohydrate reserves and maintain cellular integrity, enabling survival under prolonged hypoxia. The correlation coefficient matrix analysis (Tables 2 and 3 and Supporting Figs. 1–4) among the agronomic traits under waterlogging and recovery after drainage conditions revealed these short‐term adapting strategies. It has been reported that tolerant genotypes maintained greater shoot elongation and less reduction of shoot biomass compared with sensitive genotypes under waterlogging stress [49–51]. The observed variation in root biomass reflected genotypic differences which have been reported to involve better stress tolerance by increasing oxygenation and nutrient acquisition and transportation, CO_2_ assimilation, antioxidant enzyme activity, and the ability to maintain metabolic functions [52–55].

In the present study, tolerant jute genotypes maintained better root growth, which could be associated with efficient internal aeration through adaptive anatomical traits like aerenchyma formation, which facilitates internal oxygen diffusion from shoots to roots. Previous studies suggested that plant genotypes with enhanced aerenchyma development could maintain partial root functionality under hypoxic conditions [56, 57]. In this study, tolerant genotypes such as CVL‐1 and A‐3539 could possess greater efficiency in aerenchyma formation compared with sensitive genotypes, contributing to improved oxygenation and root survival. In addition, these tolerant genotypes could form adventitious roots for improving oxygen acquisition near the soil surface. These structural adaptations may be associated with regulating hypoxia‐induced signaling pathways for ethylene and auxin synthesis, which promote aerenchyma development, root elongation, and root architectural plasticity under waterlogging stress. Additionally, tolerant genotypes may exhibit higher activities of antioxidant enzymes, including superoxide dismutase (SOD), peroxidase (POD), and catalase (CAT), contributing to ROS detoxification.

Following drainage, the recovery phase becomes critical for plant survival and productivity. Genotypes that effectively conserve energy during stress (quiescence) could rapidly resume growth, as reflected in the strong recovery performance observed in the selected genotypes. This compensatory growth may be supported by the restoration of aerobic respiration, reactivation of photosynthesis, and efficient mobilization of stored nonstructural carbohydrates. Tolerant genotypes could maintain better carbon assimilation and partitioning, allowing rapid biomass accumulation after stress removal. In addition, efficient ROS scavenging during reoxygenation is essential to prevent oxidative injury during the recovery phase. The strong positive relationship between waterlogging tolerance and recovery ability observed in this study (Figure 10) suggested that mechanisms conferring survival under stress are closely linked to poststress resilience. The present study confirmed that jute varieties CVL‐1, A‐3539, A‐1770, and TP‐3 had higher waterlogging tolerances with strong recovery capacities. A global meta‐analysis of 217 peer‐reviewed studies on different plant species under waterlogging stress conditions revealed that recovery of agronomic, physiological, and biochemical traits after well‐drainage was closely related to survival capability due to quiescence and compensatory mechanisms [43]. Previous studies suggested that stress‐tolerant genotypes may be able to maintain higher photochemical activity, leaf greenness, and gas exchange during stress conditions than sensitive genotypes, which may alleviate the stress effects on the photosynthesis machinery and improve its recovery ability [48, 58].

Rapid postwaterlogging recovery may be driven by compensatory growth mechanisms involving growth‐related transcription factors, hormonal signaling, and restored photosynthetic activity, which together rebuild biomass and yield. Across crop species, this recovery is genetically underpinned by distinct pathways; for instance, in maize and wheat, ethylene‐responsive factors (*ZmEREB180*, *TaSAMS10*, and group VII *ERFs*) promote adventitious root formation via ethylene signaling [59, 60]; in rapeseed, phytoglobin (*BnPgb1*) supports a quiescence‐based strategy by boosting antioxidant capacity and scavenging nitric oxide [61]; and in cotton, the tolerant genotype YZ1 recovers through auxin‐driven NO biosynthesis (via *GhYUC8* and *GhNIR* upregulation) alongside suppressed ROS and ABA signaling (via *GhRBOHC* and *GhNCED2* downregulation) [41, 62], whereas sensitive genotypes (YL1 and NW1) show elevated ROS and ABA and reduced NO and auxin, resulting in oxidative damage and limited recovery [63]. Extending these mechanisms to jute, the tolerant genotypes identified in this study (CVL‐1 and A‐3539) could be proposed to recover efficiently through a similar physiological strategy, such as maintaining redox homeostasis (limited H_2_O_2_ and MDA accumulation), enhanced aerenchyma formation, elevated antioxidant enzyme activity (SOD, CAT, and POD), and increased auxin levels that sustain photosynthesis, chlorophyll retention, root vigor, and delayed senescence, collectively explaining their high recovery tolerance coefficient and superior compensatory growth after stress removal.

The significant correlations among agronomic traits and their consistent behavior under stress and recovery conditions indicated that these traits collectively reflect overall plant vigor. Traits such as shoot and root length, biomass accumulation, and root development can serve as reliable proxies for screening waterlogging tolerance. The strong interdependence among these traits, as confirmed by the correlation heatmap and PCA, resulted in a dominant first principal component (> 88% variance), representing an integrated measure of growth and tolerance rather than independent trait variation. Consequently, PC1 can be interpreted as an integrated index of growth performance and stress tolerance. Such patterns are common in morpho‐physiological studies where closely related traits respond in a coordinated manner, indicating biological redundancy rather than statistical overfitting [10, 11, 64]. Given the redundancy among traits, multivariate approaches such as PCA, MFV, and CEV provide a robust framework for genotype evaluation. The CEV indices (CEVWT and CEVRT) effectively integrate multiple traits to quantify both stress tolerance and recovery capacity. The strong agreement between PCA and the correlation heatmap, together with the consistent trends observed in CEV, indicated that these traits collectively reflect a common underlying gradient of plant vigor and stress tolerance, thereby validating the use of CEV as an integrative index for genotype evaluation under waterlogging conditions. Furthermore, the classification of genotypes through hierarchical clustering (Figure 11) highlighted the substantial variation in tolerance and recovery potential, which is essential for breeding programs.

## 5. Conclusion

Waterlogging significantly reduced seedling survival and growth in jute, with mortality increasing from 30.1% to 46.6% as stress duration extended from 10 to 14 days. Substantial genotypic variation was observed in both stress tolerance and postdrainage recovery. Key agronomic traits particularly shoot and root length and dry biomass, were strongly associated with tolerance and recovery, as confirmed by correlation analysis. The integrated indices CEVWT and CEVRT, derived from membership function analysis, proved effective for comprehensive evaluation. Based on combined metrics and cluster analysis, A‐3539, CVL‐1, TP‐3, and A‐1770 were identified as tolerant with strong recovery capacity, whereas A‐2521, O‐9897, A‐1221, A‐1119, and A‐1362 were sensitive with poorer recovery. These findings highlight the potential of integrating tolerant genotypes into breeding programs to improve waterlogging resilience and ensure stable jute production in waterlogging‐prone environments.

## Funding

This work was supported by the Grants for Advanced Research in Education (GARE), Bangladesh Bureau of Educational Information and Statistics (BANBEIS), Ministry of Education (grant number LS20222183, 2022) and Science and Technology fellowship Trust (2022), Ministry of Science and Technology, The People’s Republic of Bangladesh.

## Disclosure

The funders had no role in the design of the study; in the collection, analyses, or interpretation of data; in the writing of the manuscript; or in the decision to publish the results.

## Conflicts of Interest

The authors declare no conflicts of interest.

## Supporting Information

Additional supporting information can be found online in the Supporting Information section.

## Supporting information


**Supporting Information 1** List of Supporting Figures. Supporting Fig. 1. Correlation (Pearson’s r value) between the (A) shoot length and relative shoot length (% control), (B) root length and relative root length (% control), (C) total length and relative total length (% control), and (D) root‐to‐shoot ratio and relative root‐to‐shoot ratio (% control) under control and 14 days of waterlogging stress of 15 jute genotypes. Significant differences for a two‐tailed test are indicated: ^∗^ *p* < 0.05; ^∗∗^ *p* < 0.01; ^∗∗∗^ *p* < 0.001. Supporting Fig. 2. Correlation (Pearson’s r value) between the (A) shoot fresh weight and relative shoot fresh weight (% control), (B) root fresh weight and relative root fresh weight (% control), (C) shoot dry weight and relative shoot dry weight (% control), and (D) root dry weight and relative root dry weight (% control) under control and 14 days of waterlogging stress of 15 jute genotypes. Significant differences for a two‐tailed test are indicated: ^∗∗^ *p* < 0.01; ^∗∗∗^ *p* < 0.001. Supporting Fig. 3. Correlation (Pearson’s r value) between (A) shoot length and relative shoot length (% control), (B) root length and relative root length (% control), (C) total length and relative total length (% control), and (D) root‐to‐shoot ratio and relative root‐to‐shoot ratio (% control) under control and 14 days of recovery after drainage of 15 jute genotypes. Significant differences for a two‐tailed test are indicated: ^∗^ *p* < 0.05; ^∗∗∗^ *p* < 0.001. Supporting Fig. 4. Correlation (Pearson’s r value) between the (A) shoot fresh weight and relative shoot fresh weight (% control), (B) root fresh weight and relative root fresh weight (% control), (C) shoot dry weight and relative shoot dry weight (% control), and (D) root dry weight and relative root dry weight (% control) under control and 14 days of recovery after drainage of 15 jute genotypes. Significant differences for a two‐tailed test are indicated: ^∗^ *p* < 0.05; ^∗∗^ *p* < 0.01; ^∗∗∗^ *p* < 0.001. Supporting Fig. 5. Correlation (Pearson’s r value) between the (A) waterlogging tolerance score (WTS) and relative shoot length (% control), (B) WTS and relative root length (% control), (C) WTS and relative total length (% control), and (D) WTS and relative root‐to‐shoot ratio (% control) under 14 days of waterlogging stress of 15 jute genotypes. Significant differences for a two‐tailed test are indicated: ^∗∗^ *p* < 0.01; ^∗∗∗^ *p* < 0.001. Supporting Fig. 6. Correlation (Pearson’s r value) between the (A) waterlogging tolerance score (WTS) and relative shoot fresh weight (% control), (B) WTS and relative root fresh weight (% control), (C) WTS and relative shoot dry weight (% control), and (D) WTS and relative root dry weight (% control) under 14 days of waterlogging stress of 15 jute genotypes. Significant differences for a two‐tailed test are indicated: ^∗∗∗^ *p* < 0.001. Supporting Fig. 7. Correlation (Pearson’s r value) between the (A) recovery tolerance score (RTS) and relative shoot length (% control), (B) RTS and relative root length (% control), (C) RTS and relative total length (% control), and (D) RTS and relative root‐to‐shoot ratio (% control) under 14 days of recovery after drainage on 15 jute genotypes. Significant differences for a two‐tailed test are indicated: ^∗∗∗^ *p* < 0.001. Supporting Fig. 8. Correlation (Pearson’s r value) between the (A) recovery tolerance score (RTS) and relative shoot fresh weight (% control), (B) RTS and relative root fresh weight (% control), (C) RTS and relative shoot dry weight (% control), and (D) RTS and relative root dry weight (% control) under 14 days of recovery after drainage on 15 jute genotypes. Significant differences for a two‐tailed test are indicated: ^∗∗∗^ *p* < 0.001.

**Supporting Information 2** Supporting Table 1. List of different jute genotypes with varying mortality rates under 10, 12, and 14 days of waterlogging stress at seedling stage. Supporting Table 2. Eigenvalue, contribution rate, index weight, component matrix of the relative change (% control) of different agronomic traits after 14 days of waterlogging based on the principal component analysis. Supporting Table 3. Eigenvalue, contribution rate, index weight, component matrix of the relative change (% control) of different agronomic traits recovery after 14 days of drainage based on the principal component analysis.

## Data Availability

Data will be made available on request. For requesting data, please contact to the corresponding authors.
