# Supplementary material for: Quantifying Waterlogging Tolerance and Recovery Capacity of Jute Genotypes During Early Growth Stage Through Agronomic Trait‐Based Comprehensive Evaluation Value
Source: Scientifica (Cairo). 2026 Sep 8;2026:8323167. doi: 10.1155/sci5/8323167 (PMC13551592; doi:10.1155/sci5/8323167)
Supplement: Supplementary file 1 [file SCI5-2026-8323167-s001.docx]

Suppl. Table 1. List of different jute genotypes with varying mortality rates under 10, 12 and 14 days of waterlogging stress at seedling stage

| Waterlogging | Seedling mortality (%) | | | | | | | |
| --- | --- | --- | --- | --- | --- | --- | --- | --- |
|  | 0-20% | | 21-40% | | 41-60% | | 61-80% | 81-100% |
| 10 days | A-1175 | A-1119 | A-1192 | DP-9 | A-1327 | | A-2920 | A-1417 |
|  | A-1198 | A-1854 | A-1961 | A-1266 | A-1086 | | A-2345 | A-2541 |
|  | A-2345 | A-2521 | A-3038 | A-2381 | A-1228 | | A-1465 | A-1382 |
|  | A-2407 | A-3343 | A-3542 | TP-5 | A-1759 | | A-1965 |  |
|  | A-2645 | O-9897 | A-6072 | A-1089 | A-1193 | | Padma-1 |  |
|  | A-1166 | TP-4 | A-1241 | A-1468 | A-1522 | |  |  |
|  | A-1181 | A-1770 | A-1500 | TP-7 | A-1524 | |  |  |
|  | A-1216 | TP-8 | A-1776 |  | TP-9 | |  |  |
|  | A-1362 | A-1221 | A-2041 |  | A-1078 | |  |  |
|  | A-2095 | A-1436 | A-3538 |  | A-1173 | |  |  |
|  | A-2446 | A-3659 | O-4 |  | DP-6 | |  |  |
|  | A-3218 | CVL-1 | A-1068 |  | A-1327 | |  |  |
|  | A-3423 | DP-8 | A-1530 |  | A-1086 | |  |  |
|  | A-3539 | TP-3 | A-2522 |  | A-1228 | |  |  |
|  | JRO-524 |  | DP-10 |  | A-1759 | |  |  |
| Total | 29 | | 22 | | 11 | | 5 | 3 |
| 12 days | A-2446 | A-1216 | A-1068 | A-1175 | A-3542 | A-1530 | Padma-1 | A-1417 |
|  | A-3218 | A-3423 | A-1776 | A-1166 | A-1961 | A-3538 | A-1086 | A-1382 |
|  | O-9897 | A-3539 | DP-9 | A-1181 | TP-9 | O-4 | A-1327 | A-2541 |
|  | A-3343 | A-2645 | TP-7 | A-2095 | A-1078 | A-1266 | A-1522 | A-2920 |
|  | JRO-524 | A-1854 | A-2522 | A-2407 | A-1500 |  | A-1228 | A-1465 |
|  | A-1221 | DP-8 | A-1468 | A-1198 | A-6072 |  | A-1524 | A-2345 |
|  | A-1436 | A-1119 | A-1089 | A-2345 | A-1192 |  | A-1193 | A-1965 |
|  | TP-8 | A-1362 | TP-5 |  | A-2041 |  | A-1173 |  |
|  | A-3659 | A-2521 | DP-10 |  | A-3038 |  | A-1759 |  |
|  | CVL-1 | TP-4 | A-2381 |  | DP-6 |  |  |  |
|  | TP-3 | A-1770 |  |  | A-1241 |  |  |  |
| Total | 22 | | 17 | | 15 | | 9 | 7 |
| 14 days | A-1119 | TP-8 | A-2095 | A-3659 | A-1078 | O-4 | A-1086 | A-1382 |
|  | A-1362 | A-3539 | A-2407 | DP-8 | A-2041 | A-2522 | A-1524 | A-2541 |
|  | A-1216 | CVL-1 | A-1181 |  | TP-9 | A-1266 | A-1759 | A-2920 |
|  | A-2521 | TP-3 | A-1166 |  | A-1241 | A-1776 | A-1327 | A-1417 |
|  | A-1770 |  | A-1198 |  | A-1530 | DP-10 | A-1522 | A-1465 |
|  | A-2446 |  | A-2345 |  | A-1192 | A-1175 | A-1173 | A-2345 |
|  | O-9897 |  | A-3423 |  | A-3038 | A-1089 | A-1193 | A-1965 |
|  | A-3343 |  | A-2645 |  | DP-6 | A-1068 | A-3542 | Padma-1 |
|  | JRO-524 |  | A-3218 |  | A-1468 | TP-5 | A-6072 | A-1228 |
|  | A-1221 |  | A-1854 |  | A-3538 | TP-7 | A-1500 |  |
|  | A-1436 |  | TP-4 |  | DP-9 | A-2381 | A-1961 |  |
| Total | 15 | | 13 | | 22 | | 11 | 9 |

Suppl. Table 2. Eigenvalue, contribution rate, index weight, component matrix of the relative change (% control) of different agronomic traits after 14 days of waterlogging based on the principal component analysis

| Items | Traits | Principal component |
| --- | --- | --- |
|  |  | PC1 |
| Eigenvalue |  | 6.5 |
| Contribution ratio (%) |  | 88.1 |
| Cumulative contribution ratio (%) |  | 88.1 |
| Loading matrix of each component | Shoot length | 0.89 |
|  | Root length | 0.97 |
|  | Total length | 0.93 |
|  | Root-to-shoot ratio | 0.85 |
|  | Shoot fresh weight | 0.83 |
|  | Root fresh weight | 0.92 |
|  | Shoot dry weight | 0.94 |
|  | Root dry weight | 0.86 |
| Index weight |  | 1.0 |

Suppl. Table 3. Eigenvalue, contribution rate, index weight, component matrix of the relative change (% control) of different agronomic traits recovery after 14 days of drainage based on the principal component analysis.

| Items | Traits | Principal component |
| --- | --- | --- |
|  |  | PC1 |
| Eigenvalue |  | 7.28 |
| Contribution ratio (%) |  | 91.0 |
| Cumulative contribution ratio (%) |  | 91.0 |
| Loading matrix of each component | Shoot length | 0.96 |
|  | Root length | 0.99 |
|  | Total length | 0.98 |
|  | Root-to-shoot ratio | 0.90 |
|  | Shoot fresh weight | 0.91 |
|  | Root fresh weight | 0.97 |
|  | Shoot dry weight | 0.96 |
|  | Root dry weight | 0.98 |
| Index weight |  | 1.0 |
